# Stutter Modeling in Probabilistic Genotyping for Forensic DNA Analysis: A Casework-Driven Assessment

**DOI:** 10.3390/genes16091053

**Published:** 2025-09-08

**Authors:** Camila Costa, Érica Pereira, Sandra Costa, Paulo Miguel Ferreira, António Amorim, Lourdes Prieto, Nádia Pinto

**Affiliations:** 1 Departamento de Biologia, Faculdade de Ciências, Universidade do Porto, 4169-007 Porto, Portugal; camilac@i3s.up.pt (C.C.); 2 i3S—Instituto de Investigação e Inovação em Saúde, Universidade do Porto, 4200-135 Porto, Portugal; 3 Escola de Ciências da Vida e do Ambiente, Universidade de Trás-os-Montes e Alto Douro, 5000-801 Vila Real, Portugal; 4 Biologia, Laboratório de Polícia Científica da Polícia Judiciaria (LPC-PJ), 1169-007 Lisboa, Portugal; 5 IPATIMUP—Instituto de Patologia e Imunologia Molecular da Universidade do Porto, 4200-465 Porto, Portugal; 6 Grupo de Medicina Xenómica, Instituto de Ciencias Forenses, Universidad de Santiago de Compostela, 15705 Santiago de Compostela, Spain; 7 Laboratorio ADN, Comisaría General de Policía Científica, 28039 Madrid, Spain; 8 CMUP—Centro de Matemática da Universidade do Porto, 4169-007 Porto, Portugal

**Keywords:** probabilistic genotyping software, EuroForMix, forward stutter, back stutter, DNA mixtures, weight-of-evidence

## Abstract

**Background:** Probabilistic genotyping software has become an essential tool in forensic genetics, particularly for interpreting complex DNA mixtures. Previous studies measured the impact of considering widely divergent statistical approaches in quantifying evidence, both inter- and intra-software. At a much smaller scale, this data-driven study shows how different models implemented on distinct versions of the same tool may affect the results. Among the available tools, EuroForMix stands out as a quantitative, open-source software that models various aspects of the DNA profile, including artefacts like stutter peaks. Its freeware nature allowed the use of both versions 1.9.3. and 3.4.0, between which several updates were made, including the possibility to model both back and forward stutter, compared to only modeling back stutters inputted by the expert in the earlier version. **Methods:** A total of 156 real casework sample pairs (comprising mixtures with two or three estimated contributors and associated reference) from the Portuguese Scientific Police Laboratory were analyzed using both software versions. The same input data, containing alleles and artefactual peaks, were used to reflect operational conditions. Statistical measurements were compared and further investigated. **Results:** Most Likelihood Ratio values differed in less than one order of magnitude across versions. However, exceptions were found in more complex samples, such as those with more contributors, unbalanced contributions, or greater degradation. **Conclusions:** This work emphasizes the relevance of model selection in forensic evidence quantification, even when considering different versions of the same tool. The impact of different models in statistical evaluation depends on several factors, such as sample technical conditions, genotypic profiles, and population distribution.

## 1. Introduction

Technological advances in forensic genetics have made it possible to obtain genetic profiles from increasingly smaller quantities of DNA. However, this progress has also led to greater complexity of the samples, creating a growing need for sophisticated informatics tools to overcome the associated difficulties and weigh the genetic evidence [1,2,3]. These tools can be based on two different models, qualitative or quantitative, depending on the extent of information used to quantify the genetic evidence [4,5]. In the first ones, only the qualitative information is taken into account (i.e., presence or absence of alleles), while in the second ones, the quantitative information (i.e., the height of the observed alleles) is also considered.

Nevertheless, both models quantify the weight of evidence through the computation of a Likelihood Ratio (LR) value [6,7], which compares the probability of observing the evidence assuming two alternative and mutually exclusive hypotheses. In standard identification cases with DNA mixtures, these hypotheses are “H1 = The person of interest (PoI) is a contributor to the mixture” and, alternatively, “H2 = The PoI is not a contributor nor genetically related to any contributor of the mixture”. To perform this calculation, several parameters regarding population—allele frequencies and coancestry coefficient—and analytical factors—drop-in, drop-out, analytical threshold, and modeling of stutters—need to be considered.

Informatics tools based on a quantitative model, like STRmix™ [8] or EuroForMix [9], have become the preferred approach over the more simplistic qualitative one. They account for a large amount of data and information, allowing the modeling of artefacts, such as stutter peaks, which may be present on the electropherogram (EPG) of the evidence profile. The key advantages of these tools rely on the ability to deconvolve complex DNA mixtures, estimating genotype combinations and corresponding likelihoods [5].

A stutter peak is an artefact originating during the PCR extension phase that results from a slipped-strand mispairing. During the re-annealing of the strands (template and extending strand), one may loop and be aligned in a position different from the supposed one. The more common is for the loop to occur in the template strand, resulting in the deletion of one (or more) repeat units in the new strand—the so-called back stutter, whose height corresponds to a considerable proportion of the allelic peak height (5–10%). Even though it is less common, the loop can also occur in the new strand, leading to the addition of repeat unit(s) in the new strand—forward stutter, resulting in a peak that accounts for a smaller fraction of the parent allelic height (0.5–2%) [10,11,12].

This artefact is one of the most observed in EPG and may be challenging to distinguish from alleles from minor donors, especially in mixed-source samples, and can lead to an inaccurate estimation of the number of contributors. Also, different studies emphasize that, to maximize the statistical significance of the LR value, stutter peaks should be included in the analysis and appropriately modeled, rather than applying a stutter filter (during the STR genotyping phase) or relying on (subjective) human decisions in what is a stutter or a true allele peak. These studies further reinforce that the amplification product of an allele is a combination of both true allele copies and their associated stutter peaks, which should not be disregarded [13].

For these reasons, some quantitative tools such as STRmix™ [8] require the inclusion of stutter peaks in the analysis [13] and model them by considering expected stutter ratios per locus derived from empirical data. Others, like EuroForMix [9], let the user decide whether to model stutters, applying a general expected stutter rate as an extension of the probabilistic model [14]. The effort and dedication to include and analyze this parameter have been evident over time. For example, in early versions of EuroForMix, as version 1.9.3, it was only possible for the expert to choose model back stutters. The more recent versions, as v. 3.4.0, also support the modeling of forward stutters (available at: https://www.euroformix.com/, accessed on 3 April 2023). Despite this major improvement, it is noteworthy that between these versions, EuroForMix underwent other algorithmic improvements, especially related to optimization strategies. Previous research has compared widely differing statistical architectures and models used in various software tools, and even within the same tool. It has already been shown that distinct statistical frameworks—including those for DNA quantity and parameter estimations—can lead to substantially different results for the same input data [15,16,17,18]. However, a finer-scale comparison of modeling differences between versions of the same tool, specifically regarding the stutter model, has never been attempted.

This work aims to demonstrate the implications of analyzing the same input data under different stutter modeling options: modeling either back or both back and forward stutters by using different versions of the same software. This methodology was selected to replicate real-world forensic workflows where entire software versions are replaced rather than individual features switched. To accomplish this, pairs of real casework samples composed of a mixture (with two or three estimated contributors) and an associated reference were analyzed using the two EuroForMix versions abovementioned (1.9.3 and 3.4.0), as the older version only enables the expert to input whether to model back stutter, while the newer enables modeling both back and forward stutters.

## 2. Materials and Methods

In this study, real casework samples were used, rather than mocked ones, to more authentically replicate the complex and unique conditions that forensic experts encounter during routine genetic analyses. A total of 156 irreversibly anonymized DNA pairs of mixtures (78 with two and 78 with three contributors previously estimated by independent analysts) and an associated single-source profile were selected from former cases of the Portuguese Scientific Police Laboratory of the Judiciary Police. All samples were previously processed in the context of the respective casework, under manufacturer’s and internal protocols, and all methods were carried out following relevant guidelines and regulations. The genetic information of each selected pair analyzed in this work was amplified using the GlobalFiler™ PCR Amplification Kit (Applied Biosystems™, Thermo Fisher Scientific Inc., Waltham, MA, USA) and GlobalFiler™ Express PCR Amplification Kit (Applied Biosystems™, Thermo Fisher Scientific Inc., Waltham, MA, USA), both 24-locus STR kits with an analytical threshold equal to 100 RFU. A set of 21 autosomal STR markers was considered, coinciding with the data analyzed in [15,17]. Proper ethics approval was obtained from the Committee for Ethical and Responsible Conduct of Research of i3S—Instituto de Investigação e Inovação em Saúde, Universidade do Porto, Portugal (5/CECRI/2022) concerning all the statistical analyses.

To conduct this study, two versions of the quantitative software EuroForMix [9] were used: v.1.9.3 and 3.4.0, as they incorporate different approaches to stutter modeling—the first allows only the modeling of back stutters, while the updated version allows the modeling of both back and forward stutters. These were the features correspondingly selected for the analysis. As is the standard methodology for comparative analyses, the same input profiles—containing alleles and artefactual peaks, including back and forward stutters– were used in both tools. This also reflects a typical operational scenario, where casework profiles may contain artefacts—indistinguishable from true alleles, unmodeled by the software used.

Through the comparison of the results obtained by each software version, it is possible to evaluate the impact of software modeling updates when quantifying the same input data. All the remaining parameter settings were held constant across versions, as detailed in Supplementary Table S1. For each version and selected pair of samples, LR values were calculated assuming the alternative hypotheses: H1—“The PoI is a contributor to the mixture” versus H2—“The PoI is not a contributor and is not genetically related to any contributor of the mixture”. The reference sample used was assumed to belong to the PoI. Following recommendations from the software developers [9], the Maximum Likelihood Estimation (MLE) was employed in both versions for model comparison. To perform the calculations, the allele frequencies of the National Institute of Standards and Technology (NIST) database concerning the Caucasian population were used [19]—see Supplementary Table S2.

After completing all calculations, a comparison of the LR values assigned by both versions was performed for each pair of samples using their ratio: R = LR1/LR2 if LR1 > LR2, or R = LR2/LR1, otherwise. Thus, for example, a value of 1 < R < 10 indicates that one LR (either obtained using only back stutter modeling or both back and forward stutters) is at most ten times greater than the other. The level of complexity of the mixture samples analyzed was assessed based on two parameters estimated under H1—the contributors’ mixture proportion and the degradation slope. Through the first one, it is possible to understand how imbalanced the contributions are; while the second reflects the level of degradation, typically ranging between 0 and 1, with 1 corresponding to no degradation and values below 0.60 to very degraded samples [20].

Diagnostic metrics estimated under H1 and associated with the LR value, such as Loglik and Peak Height Variability, were also assessed. Both values allow the expert to understand if the model fits the observed data: the higher the Loglik value, the better the model fit; on the other hand, a lower Peak Height Variability means a better description of the model. Statistical tests were performed to assess the paired differences in the results obtained from the different software versions. The Shapiro–Wilk test was used to assess data normality. Subsequently, if normality could be assumed, the differences between the corresponding means were evaluated using a Paired T-test; otherwise, the Wilcoxon Signed-Rank test was applied. All analyses were performed using functions from the R software v.4.1.2. Additionally, a chi-square goodness-of-fit test was conducted to infer whether there was a significant increasing or decreasing trend, assuming an equal number of decreasing and increasing LRs as the expected distribution. A significant level of a = 0.05 was used for all the statistical analyses.

## 3. Results and Discussion

**Figure 1:**
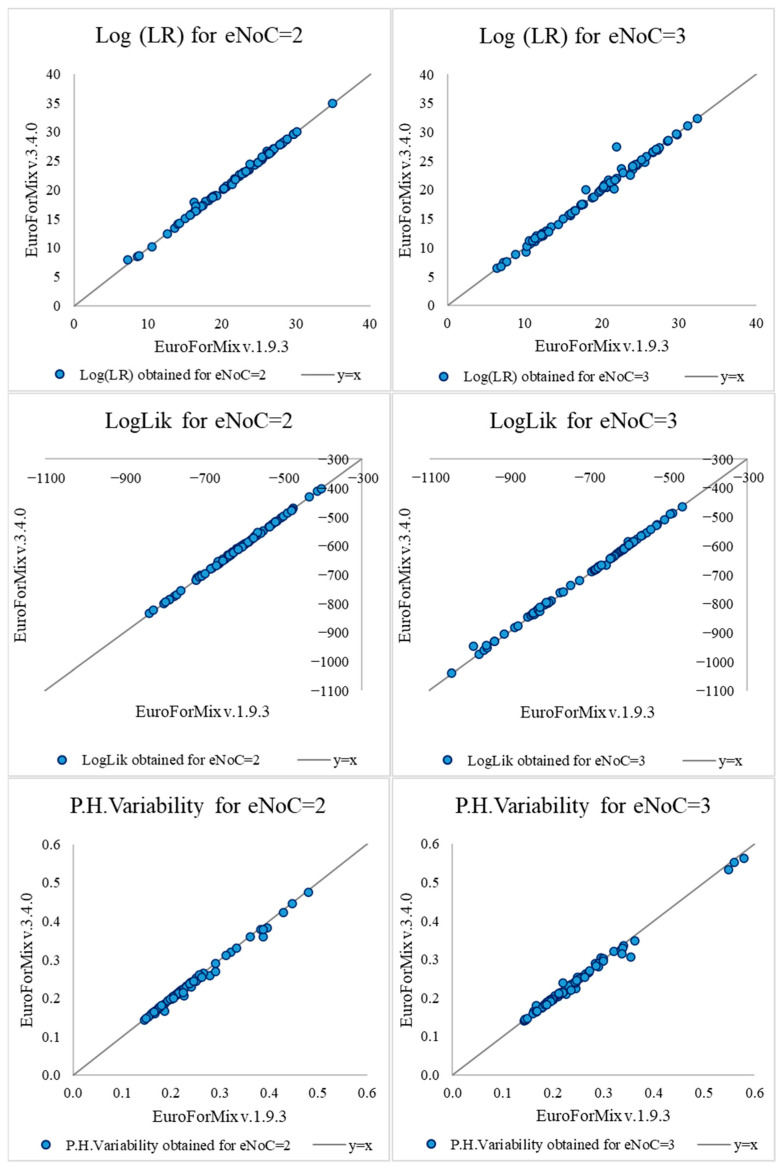
Distribution of LRs and associated diagnostic values, LogLik and Peak Height Variability (estimated under H1) obtained for both EuroForMix versions (1.9.3 in *x*-axis and 3.4.0 in *y*-axis), for both cases with mixtures with two (left plots) and three (right plots) estimated contributors. The order of pairs of samples is the same in each plot and corresponds to the same data displayed in Supplementary Material Table S3. For example, the first dot in each plot corresponds to pair 1_eNoC, in which eNoC (estimated number of contributors) is either 2 or 3 if considering the left or right plots, respectively.

LR values obtained assuming only back (EuroForMix v.1.9.3) and both back and forward stutter modeling (v.3.4.0) were compared for mixtures with both two and three estimated contributors—see Supplementary Table S3 for the complete set of LR results and corresponding diagnostic values and Figure 1 for their distribution. The differences observed between LR values are presented in Table 1.

In Supplementary Table S3, other estimations performed under H1 are present as well for all the analyses and samples. These include contributors’ mixture proportions, expected peak height, peak height variability, and degradation slope. Notably, the complexity of the mixture samples analyzed, for both mixtures with two and three estimated contributors, becomes evident in these parameters. Indeed, using v.1.9.3, PoI was considered the minor contributor in 32 of the 78 analyzed cases with two estimated contributors (under H1, estimated proportion of contribution: 0.0937 to 0.4694, median = 0.3238, and a mean = 0.3112), and in 3 out of the 78 cases with three estimated contributors (under H1, estimated proportion of contribution: 0.1025 to 0.3161, median = 0.2365, and a mean = 0.2137). On the other hand, the estimated degradation slope provides insight into the degree of sample degradation. For both two and three estimated mixture contributors, the degradation slope medians were similar (values estimated under H1, 0.7510 and 0.7434, respectively), and the means were also close (0.7389 and 0.7252, respectively). Notably, 25% of the samples in both groups had estimated slopes of 0.6498 (two contributors) and 0.6367 (three contributors), values close to the 0.60 threshold considered typical for highly degraded samples. For version v.3.4.0, similar parameter values were observed for both cases with mixtures with two and three estimated contributors.

As illustrated in Table 1, for most cases with mixtures with two and three estimated contributors (99% and 95%, respectively), the LR values assigned by both software versions were within the same order of magnitude—that is, for these cases the LR differences modeling both back and forward stutters or only modeling back did not exceed a factor of ten. Despite no statistically significant differences between median LR difference results being found for both mixtures with two (*p*-value = 3.89 × 10^−01^, Wilcoxon Signed-Rank test; *p*-value = 2.53 × 10^−12^, Shapiro–Wilk Test) and three estimated contributors (*p*-value = 4.40 × 10^−01^, Wilcoxon Signed-Rank test; *p*-value = 2.67 × 10^−15^, Shapiro–Wilk Test), some cases exhibited large differences, particularly for mixtures with three estimated contributors. This is expected, as mixtures with three contributors may have more complexity associated and exhibit more pronounced effects from artefacts such as forward stutters, especially if not explicitly modeled. Indeed, from the 78 pairs of samples analyzed with mixtures with two estimated contributors, only one showed LR differences greater than ten times (R = 3.70 × 10^01^), raising this number to four in the case of mixtures with three estimated contributors (R = 1.32× 10^01^, R = 1.39 × 10^01^, R = 1.64 × 10^02^, and R = 3.34 × 10^05^).

Unsurprisingly, these cases correspond to particularly complex mixtures and sensitive situations that stand out not only due to the presence of unbalanced contributors and degraded samples but also because the genotypic configurations (often involving rare alleles) are difficult to explain under the stringency of the stutter model. For illustrative purposes, special attention was devoted to the case with the most striking difference observed (R = 3.34 × 10^05^). Through a thorough per-marker analysis of this case with a mixture of three estimated contributors, most showed modest differences between software versions, with LR ratios ranging from 0.36 to 1.73, and consistently supported the same hypothesis across versions. In contrast, two markers (D21S11 and D18S51) stood out not only because different versions supported different hypotheses—the older version supported H2, while the recent version supported H1—but also due to the substantially higher ratios obtained (501.53 and 228.79, respectively)—see Supplementary Material Table S4. Thus, these two loci were mainly responsible for the largest discrepancies observed. At D18S51, the mixture showed alleles 13 (232 RFU), 14 (160 RFU), 15 (2101 RFU), 16 (139 RFU), and 17 (1820 RFU), while the reference was homozygous 15,15. The pronounced imbalance and presence of several minor peaks made interpretation difficult. In version 1.9.3, the extra peaks and their respective heights were difficult to justify due to modeling limitations in explaining the genotypic observations, given the 8:1:1 mixture ratio with the PoI as the major contributor. These difficulties lowered the likelihood under Hp and resulted in a low LR value (0.088). In version 3.4.0, the model incorporated these peaks more efficiently–some, such as peak 16, could have been modeled as forward stutters–along with other algorithmic improvements. These changes allowed the reference genotype to better explain the evidence profile, substantially increasing the LR (20.20). This demonstrates the locus’s sensitivity to interpretative assumptions in mixtures. At D21S11, the effect was even more pronounced. The evidence showed alleles 27 (293 RFU), 28 (2367 RFU), and 29 (2356 RFU). Given the 8:1:1 mixture ratio with the PoI as the major contributor, the strong 29 signal is fully consistent with the reference profile (29, 29), and 28 can be readily attributed to a minor contributor. The key interpretative challenge is the smaller allele 27, which complicates the profile despite its lower height. In the earlier version, despite already modeling back stutter, allele 27 was treated as a true allele from an unknown contributor, most likely because its relative peak height did not match well with expected stutter proportions. Given its low frequency (0.02), this assignment heavily penalized the probability of Hp, resulting in a very low LR (0.028). The updated version was able to accommodate allele 27 more consistently as a back stutter from 28, avoiding the need to assign it as a genuine allele of a minor contributor. This improvement raised the LR to 14.18 and shifted the balance of evidence in favor of Hp. Together, these loci illustrate how features like allelic imbalance, additional low peaks, but also rare alleles in the population can make results highly sensitive.

On the other hand, when analyzing the diagnostic values associated—Loglik and Peak Height Variability—statistical evidence seems to support better modeling of observed data by the latest version. Statistically significant differences between median of results were found for both mixtures with two (*p*-value = 7.00 × 10^−09^ and 1.54 × 10^−08^, Wilcoxon Signed-Rank test, respectively; *p*-value = 0 and 3.16 × 10^−12^, Shapiro–Wilk Test, respectively) and three estimated contributors (*p*-value = 5.26 × 10^−12^ and 7.98 × 10^−07^, Wilcoxon Signed-Rank test, respectively; *p*-value = 1.06 × 10^−13^ and 1.96 × 10^−10^, Shapiro–Wilk Test, respectively). The Loglik tended to increase when both back and forward stutters were modeled (*p*-value = 1.90 × 10^−07^ and 2.22 × 10^−12^, respectively, chi-square test), and the Peak Height Variability tended to decrease (*p*-value = 5.92 × 10^−06^ and 6.29 × 10^−07^, resp., chi-square test) for both cases with two and three estimated contributors. Nevertheless, caution should be taken when generalizing these results for several reasons, from both theoretical and practical points of view. Regarding the first, it is noteworthy that increasing the number of parameters in a statistical model inevitably increases the degrees of freedom, which necessarily impacts the diagnostic values and corresponding model fit. From a practical, methodological point of view, it is essential to note that the same input files were used in both software versions, thereby replicating the standard approach employed in both comparative analyses and laboratory procedures when software updates are installed. In the scope of this work, depending on the case under analysis, this may have important consequences for the diagnostic values of the earlier version, which only considers backward stutters. This scenario is easy to explain—if the longer peak observed in the data is, in fact, a forward stutter (likely with a small DNA quantity), then models that do not account for forward stutters might incorrectly consider it as an allele. This misclassification could lead to skewed diagnostic values, where the low peak height influences the model’s interpretation, potentially compromising the accuracy of the results.

## 4. Conclusions

The implementation and use of probabilistic genotyping tools in forensic genetics has increased over the past few years, enabling experts to quantify the weight of highly complex genetic samples encountered in casework. Software based on quantitative models has become the preferred approach over the more simplistic qualitative one, as it accounts for a large amount of data and information, also modeling artefacts, such as stutter peaks. Nevertheless, despite their advantages, these tools are still often perceived as black boxes by forensic geneticists, as the mathematical and statistical models incorporated are beyond the typical user’s expertise. This complexity (and consequent understanding) increases as tools evolve to handle more detailed information from increasingly complex samples. Thus, using a data-driven approach, the main goal of this paper is to demonstrate the impact that applying different modeling approaches to the same input data may have, even using the same software. As software versions advance and new modeling capabilities are introduced, such as the ability to include forward stutters, it becomes crucial to understand how these developments may influence the interpretation of forensic evidence.

Staying true to the complexity of DNA samples encountered in real forensic casework, it is impossible to mimic with mocked ones; exclusively, real casework data were analyzed in this study. Each data pair consisted of a DNA mixture (with either two or three estimated contributors) and an associated reference profile. The selected samples were processed using two different versions of the same quantitative informatics tool (EuroForMix v.1.9.3 and 3.4.0). The main difference between the two versions lies in the modeling of stutter peaks—the first is only able to model back stutters, while the latter models both back and forward stutters.

By comparing LR results obtained for each pair of samples, along with the associated diagnostic values (Loglik and Peak Height Variability), it was possible to observe that most LR values remained within the same order of magnitude between the two software versions. Diagnostic values tended to indicate improved model fit when both back and forward stutters were included, as evidenced by generally higher Loglik values and lower Peak Height Variability values. However, it is important to interpret these findings cautiously. The inclusion of forward stutters modeling introduces additional parameters, thereby increasing the model’s degrees of freedom, which naturally tends to improve fit diagnostics. Consequently, the better diagnostic performance of the latest software version should not be taken as definitive proof of superior modeling, but rather as an indication that more complex models may capture additional data features. Furthermore, the consistent use of identical input files across software versions ensures comparability but also highlights potential misclassifications in the earlier model that only considers back stutters, leading to forward stutter peaks being mistakenly classified as alleles, especially in low DNA quantity samples, potentially biasing diagnostic values. Thus, while the results suggest advantages in modeling both stutter peaks, the interpretation must be balanced with an understanding of the underlying model complexity and the biological and technical context of each case.

This work provides a practical example of how changes in modeling strategies—implemented even within the same tool—can influence the statistical results regarding the quantification of the observations in the forensic genetics setting. The use of more comprehensive models allows the software to better address the inherent complexity of the real casework data, as illustrated in this study. It also reinforces the importance of laboratories regularly updating the software used in their workflow, as ongoing developments contribute to improving accuracy, transparency, and reliability in forensic DNA interpretation. Therefore, it is strongly recommended that forensic laboratories not only validate updated software versions prior to implementation but also remain aware of the interpretive implications such changes may bring, particularly in complex or borderline cases.

## Figures and Tables

**Table 1:** Ratio R (R > 1) between LR values computed through EuroForMix v.1.9.3 (only back stutter modeled) and v.3.4.0 (back and forward stutters modeled) for mixtures with two and three estimated contributors; the mean, median, and maximum LR values are also described. The evidence of a general trend for the LR values to increase or decrease when using the more recent version is presented. * See Supplementary Table S3 for all LR values assigned with both software versions and respective diagnostic values.

**Ratio Between LRs** **(v.1.9.3 vs. v.3.4.0, R > 1)**	**Estimated Number of Contributors**	
	**2**	**3**
1 < R < 10	99%	95%
10 < R < 100	1%	3%
100 < R < 1000	0%	1%
1000 < R < 10,000	0%	0%
R > 10,000	0%	1%
Mean	0.1244	0.2794
Median	0.0400	0.1034
**Maximum Ratio Between LRs**		
R	3.70 × 10^01^	3.34 × 10^05^
LR (v.1.9.3)	1.70 × 10^16^	7.93 × 10^21^
LR (v.3.4.0)	6.28 × 10^17^	2.65 × 10^27^
Pair *	24_2	7_3

## Data Availability

All the statistical analyses supporting the findings of this study are included in the Supplementary Material. The raw genotypic and haplotypic data are derived from real forensic casework from the Portuguese Scientific Police Laboratory, Judiciary Police (LPC-PJ), and therefore cannot be publicly released. However, non-haplotypic data related to specific analyzed markers may be available upon reasonable request and with prior permission of LPC-PJ. For inquiries regarding data access, contact the corresponding author of the manuscript.

## Supplementary Materials

The following supporting information can be downloaded at: https://www.mdpi.com/article/10.3390/genes16091053/s1, Table S1: Software parameters values and conditions considered in both EuroForMix versions used in this work: 1.9.3 and 3.4.0. Table S2: Allele frequencies of the National Institute of Standards and Technology (NIST) database considered. Table S3: LRs: diagnostic values, mixture proportions, and degradation slope (all estimated under H1) obtained from both EuroForMix versions (1.9.3 and 3.4.0), for both cases with mixtures with three estimated contributors. Table S4: LR values (overall and per-marker) and diagnostic values obtained from both EuroForMix versions (1.9.3 and 3.4.0), for the pair 7_3, the one with the biggest discrepancy between versions. The genotypic data of both the evidence and reference profiles are also provided.
