# Supplementary material for: Stutter Modeling in Probabilistic Genotyping for Forensic DNA Analysis: A Casework-Driven Assessment
Source: Genes (Basel). 2025 Sep 8;16(9):1053. doi: 10.3390/genes16091053 (PMC12469979; doi:10.3390/genes16091053)
Supplement: Supplementary file 1 [file genes-16-01053-s001.zip › Table S1_software parameters considered.pdf]

Table S1: Software parameter values introduced in both versions (1.9.3 and 3.4.0) of the quantitative tool EuroForMix.

Parameters	Values
**Drop-in frequency**	0.05
**Drop-in parameters' distribution (λ)**	0.01
**Analytical Threshold**	100
**Coancestry coefficient**	0.01
